# The innovation of traditional handicrafts and cultural identity: A multidimensional value analysis using the DEMATEL-ISM method

**DOI:** 10.1371/journal.pone.0322893

**Published:** 2025-05-08

**Authors:** Hong Li, Tao Xue, Zhong Zheng, Xuexing Luo, Guanghui Huang

**Affiliations:** 1 Faculty of Humanities and Arts, Macau University of Science and Technology, Taipa, Macau, China; 2 Zhuhai M.U.S.T. Science and Technology Research Institute, Zhuhai, Guangdong, China; University of Economics Ho Chi Minh City, VIET NAM

## Abstract

This study aims to explore how cultural identity influences the protection and innovation of traditional crafts through multidimensional pathways. Using the examples of macau shipbuilding and portuguese tile painting, 14 influencing factors were extracted from three dimensions—culture and expression, aesthetics and creation, and cognition and emotion—through the Delphi method and expert feedback. The decision-making trial and evaluation laboratory (DEMATEL) method was used to analyze causal relationships, and interpretive structural modelling (ISM) was employed to construct a hierarchical model consisting of core, direct, and indirect factors. The results indicate that historical continuity, knowledge transmission, and the integration of tradition and modernity are core factors, forming the cultural foundation for traditional craft innovation. Direct factors, such as creative expression, local culture, and emotional communication, play a pivotal role in connecting the core and indirect layers, while indirect factors like visual appeal, design originality, and craft education reflect the multidimensional value of traditional crafts. The study provides a clear, hierarchical pathway that explains the systematic process from cultural identity to innovation practice, offering valuable insights for sustainable craft innovation in the context of globalization.

## 1 Introduction

As globalization and modernization intensify at an accelerating pace, the transmission and innovative transformation of cultural heritage have emerged as critical global concerns. In recent years, traditional crafts have reclamation of prominence, driven by a renewed appreciation for their distinctive uniqueness and the intrinsic value of handmade artistry [1]. Amid challenges such as raw material scarcity, escalating manufacturing costs, skill deficiencies, and regulatory constraints on usage, innovation has proven indispensable for the sustained preservation of these crafts [2]. As vital conduits of regional or national culture, traditional handicrafts encapsulate profound historical significance and distinctive aesthetic principles, serving not merely as expressions of cultural identity but also as catalysts for social cohesion and creative ingenuity [3]. Cultural identity, defined as the sense of affiliation and recognition that individuals or collectives associate with their culture, history, values, customs, and beliefs [4], Cultural identity is not only the core value of handicrafts [5]. Moreover, it provides an essential criterion for assessing their artistic merit, historical importance, and market viability [6,7].

Macau, as a significant city where eastern and western cultures converge, boasts a unique geographical and cultural background. It is deeply rooted in traditional Chinese culture while also influenced by the portuguese colonial period. Its traditional crafts integrate the characteristics of both chinese and portuguese cultures, making it an ideal case for studying the relationship between cultural identity and craft innovation. Macau’s shipbuilding craftsmanship, with its strong ties to fishing traditions, and Portuguese tile painting, with its distinctive artistic style, reflect the intertwining of colonial and local cultures. These two crafts are key representations of macau’s cultural identity. Today, traditional handicrafts in macau face the following dilemmas: on one hand, balancing cultural preservation with commercial development; on the other, enhancing the cultural identity and market competitiveness of traditional handicrafts through innovative design. The production of traditional handicrafts has increasingly become mechanized [8] and culturally homogenized [9]. Existing research on the innovation of traditional crafts remains insufficient, especially regarding how to balance the inheritance of traditional skills with innovation [10]. Moreover, constructing a systematic analytical framework to reveal the mechanisms through which cultural identity influences craft innovation remains an area worthy of further exploration.

This study selects macau’s shipbuilding craftsmanship and Portuguese tile painting as case studies. By constructing a scientific and systematic indicator system, it aims to reveal how cultural identity influences the protection and innovation of traditional crafts through different pathways. The main research steps include: (1) identifying key indicators of cultural identity’s influence on traditional crafts through the delphi method and literature review; (2) using the DEMATEL method to identify key factors and causal relationships; and (3) constructing a multi-level model based on the ISM method to clarify the role and pathways of cultural identity in craft innovation. This study combines DEMATEL and ISM, proposing an analytical framework that can serve as a reference for similar research on cultural identity and craft innovation in other regions. This integrated methodological approach affords robust exploratory capacity and complementarity in addressing complex systems, delivering a multidimensional analytical lens. It not only elucidates the fundamental driving forces underpinning the innovation of traditional craftsmanship in Macau but also furnishes substantive support and a referential framework for investigations into craftsmanship innovation and cultural identity across diverse regional contexts.

## 2 Extraction of influencing factors

**Fig 1:**
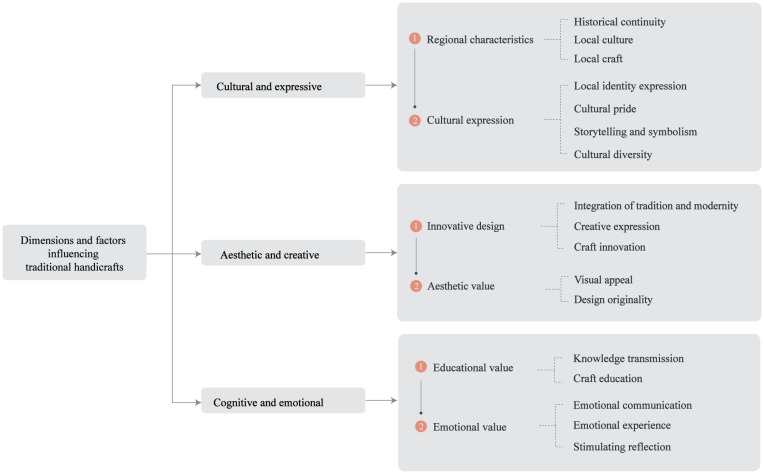
Conceptual diagram of dimensions and factors influencing traditional craft innovation.

To formulate the item pool for this study, the investigation centered on an exhaustive review of extant literature pertaining to traditional craftsmanship innovation and cultural identity, through which critical influencing factors were discerned. Within the domain of traditional craftsmanship, artifacts crafted via manual techniques and time-honored methods embody distinct regional, historical, and cultural symbolism, frequently intertwined with the social, economic, and cultural fabric of a specific locale [11,12]. In cultural identity, individuals or groups strengthen their sense of cultural belonging through participation in and expression of traditional cultural activities, such as festivals and artistic creation, which are reflected through social interactions and cultural practices [13,14]. These items cover three primary indicators—Culture and expression [15], Aesthetics and creativity [16], and Cognition and emotion [17,18]—which are further divided into six secondary indicators, 17 tertiary indicators, and 34 specific items, thus forming the preliminary framework and content for the item pool (see Table 1). Fig 1 illustrates the conceptual structure diagram of the dimensions and their associated factors.

**Table 1:** Dimensions and factors influencing traditional craft innovation from cultural identity perspective.

First-level indicator	Description	Second-level indicator	Description	Third-level indicator
Cultural and expressive (AB)	Focusing on how traditional crafts reflect local culture, historical heritage, and expressions of cultural identity.	Regional characteristics (A)	The uniqueness of local culture and history, emphasizing the deep roots of traditional crafts in regional cultural identity.	Historical continuity
				Local culture
				Local craft
		Cultural expression (B)	Crafts convey local cultural narratives through forms, designs, and symbolism, stimulating an understanding and connection to regional culture.	Local identity expression
				Cultural pride
				Storytelling and symbolism
				Cultural diversity
Aesthetic and creative (CD)	Exploring the expression of traditional crafts in innovation and aesthetics, emphasizing the fusion of artistry and creativity.	Innovative design (C)	In modern society, traditional crafts, through innovation and transformation, integrate contemporary design thinking, resulting in novel forms of expression and functionality.	Integration of tradition and modernity
				Creative expression
				Craft innovation
		Aesthetic value (D)	Evaluating the artistic appeal of traditional crafts from a visual and perceptual perspective, focusing on the aesthetic qualities of the craftwork and the uniqueness behind its creation.	Visual appeal
				Design originality
Cognitive and emotional (EF)	Emphasizing the impact of traditional crafts on knowledge transfer and emotional resonance, and their role in education and emotional communication.	Educational value (E)	Enhancing societal awareness and understanding of traditional crafts through the study and dissemination of craft knowledge and techniques.	Knowledge transmission
				Craft education
		Emotional value (F)	Focusing on the role of crafts in emotional expression and communication. It is not only an artwork but also a medium for conveying emotions.	Emotional communication
				Emotional experience
				Stimulating reflection

### 2.1 Cultural and expressive factors

(1) Regional characteristics: Regional characteristics manifest through the synthesis of local culture, historical continuity, and regional crafts, with handicrafts functioning as conduits of cultural memory and social emblems for the region. Historical continuity denotes the capacity of handicrafts to exhibit historical culture and perpetuate it as cultural heritage [19], such as the transmission of traditional skills, the reproduction of cultural elements, and the passing of techniques between generations [20,21]. Local culture encapsulates the distinctive cultural attributes of specific regions, encompassing regional symbols, customs, beliefs, and values [22]. For instance, Suzhou embroidery, through its fusion of traditional craftsmanship with local historical culture, emerges as an emblem of regional identity and societal values [23]. Local craft refers to the traditional materials, techniques, and forms used in specific regions, embodying the unique cultural expressions of the area [24]. Through material selection, craftsmanship techniques, and the forms of handicrafts, local craft becomes a carrier of regional cultural characteristics [19].
(2) Cultural expression: Cultural expression pertains to the articulation of local culture, history, and diversity through handicrafts [12], encompassing primarily the manifestation of local identity, the reinforcement of cultural pride, the transmission of narratives and symbolism, and the integration of multicultural elements. Local identity expression refers to how handicrafts, as cultural carriers, embody and convey local identity and social characteristics [25]. During traditional festivals, the exhibition of handicraft activities amplifies the distinctiveness of local culture [26]. Cultural pride is fortified through the creation and presentation of handicrafts, cultivating a profound sense of belonging and identity among individuals and communities [27], whereas the erosion of traditional crafts, precipitated by diminished market demand, may engender a concomitant loss of cultural pride [28]. Storytelling and symbolism refer to the replication of specific cultural narratives or historical episodes within handicrafts, rendering these artifacts repositories of cultural memory and historical discourse [29]; yet, such expressions are frequently distilled into commercial symbols, thereby sacrificing their cultural profundity. Cultural diversity signifies the assimilation of varied cultural elements into traditional handicrafts, exemplifying their capacity for inclusivity [30]. For example, Kyoto kimono production in Japan combines natural perceptions with traditional dyeing and weaving techniques, becoming a cultural symbol that blends both traditional and modern elements [31].

### 2.2 Aesthetic and creative factors

(1) Innovative design: Innovative design encompasses the synthesis of tradition and modernity, creative expression, and craft innovation, safeguarding cultural values while facilitating adaptation to contemporary societal needs [32]. The integration of tradition and modernity entails the incorporation of modern design principles and technological advancements while upholding traditional techniques and cultural significance, thereby enabling traditional handicrafts to align with the exigencies of the contemporary marketplace [33]. For instance, the amalgamation of traditional embroidery with modern fashion design augments its market competitiveness, whereas traditional crafts devoid of modern design elements risk marginalization within the market [34]. Creative expression pertains to innovation in the form, materials, and functionality of handicrafts to address the preferences of modern consumers [35], with handicrafts lacking such ingenuity potentially forfeiting their market relevance. Craft innovation denotes technological advancements and refinements in craftsmanship grounded in traditional skills, aimed at enhancing production efficiency and artistic articulation [36]. For example, the application of digital technology has elevated the production efficiency of traditional crafts; however, the absence of adequate technical and financial resources may impede innovation, underscoring the necessity for societal and governmental support [37,38].
(2) Aesthetic value: This emphasizes the importance of visual appeal and design originality. Visual appeal refers to the aesthetic impact of handicrafts in terms of color, form, and patterns, which can attract consumers’ attention and influence their market acceptance [39]. Design originality refers to the uniqueness of handicrafts in artistic and cultural aspects, giving them irreplaceable value in the market [40]. Design originality is not only a competitive advantage for handicrafts in the market but also enhances their recognition and brand influence by highlighting their unique cultural connotations [41]. For example, Chinese Miao embroidery strengthens its artistic value and cultural recognition through innovative pattern designs, gaining higher market recognition [42].

### 2.3 Cognitive and emotional factors

(1) Educational value: Educational value pertains to the perpetuation and advancement of traditional craftsmanship through the dissemination of knowledge and the provision of skill-based education. Knowledge transmission encompasses the impartation of the history, culture, and techniques associated with handicrafts to the public via exhibitions, courses, and other educational modalities [43]. Local community workshops and university curricula focused on intangible cultural heritage enable younger generations to comprehend and acquire mastery over traditional crafts through a blend of theoretical instruction and practical engagement [44,45]. Craft education focuses on teaching traditional skills through formal or informal educational methods [17]. The sustainability of craft education hinges on market demand and societal emphasis on traditional culture, which collectively determine the efficacy with which traditional skills are transmitted across generations [46].
(2) Emotional value: Emotional value refers to the way in which handicrafts deepen their social significance through emotional communication, emotional experience, and stimulating reflection. Emotional communication refers to the emotional connection established between the maker, user, and viewer through the creation or appreciation of handicrafts [47]. The unique details and handmade traces on a craft can convey the maker’s emotions, allowing consumers to feel the creator’s intention and the warmth of their craftsmanship [48,49]. Emotional experience underscores the affective resonance of handicrafts for both the maker and the user [18], whereby prolonged cultural engagement may elicit deep emotional responses from consumers. Stimulating reflection refers to the capacity of handicrafts’ cultural connotations and artistic expressions to prompt viewers to engage in profound contemplation of history, culture, and personal ways of life [50]. For instance, bamboo weaving integrates cultural narratives into its design, allowing consumers to not only appreciate the product’s practicality but also reflect on the relationship between modern life and traditional culture [51].

## 3. Research methodology

### 3.1 Research background

**Fig 2:**
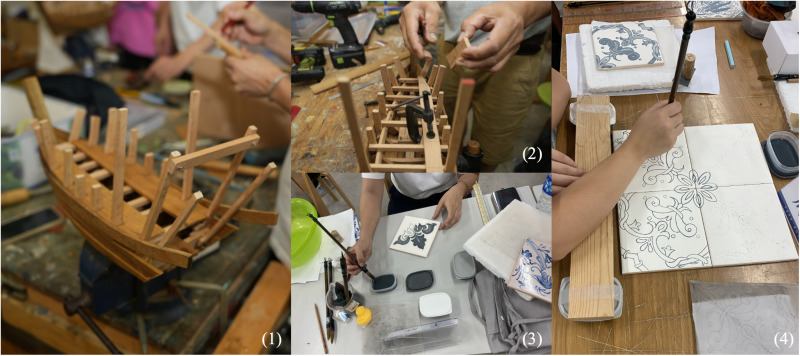
On-site training of the project, where (1) and (2) are Macao shipbuilding handicrafts, and (3) and (4) are Portuguese ceramic tile painting handicrafts.

Macau, with its unique geographical location and diverse culture, integrates various cultural influences, including chinese and portuguese traditions. Traditional handicrafts are an important expression of this cultural fusion. Macau’s shipbuilding craftsmanship is another vital tradition. As a harbor city where eastern and western cultures converge, Macau has a century-long history of shipbuilding, once one of its four major traditional industries [52]. At its peak, Macau had over 30 shipyards, which used traditional shipbuilding methods passed down through generations. Today, the old shipyards still showcase these traditional techniques. Macau’s shipbuilding craftsmanship is not only a cultural heritage but also a testament to the wisdom and labor of its artisans. The Portuguese tile painting, Azulejo, a term derived from arabic meaning “polished little stone” [53], was used by the portuguese for architectural decoration, often featuring geometric and plant patterns. This style is commonly found in macau’s street signs, buildings, and interior decorations, and artists still create tile paintings for use in architecture and souvenir design [54]. This study is based on a certain training topic, which underwent a rigorous selection process to appoint 20 eminent experts from a pool exceeding 200 candidates nationwide. These specialists possess extensive expertise in handicrafts, cultural heritage preservation, and innovation. The project emphasized professional training in Macau’s shipbuilding craftsmanship and Portuguese tile painting, as shown in Fig 2.

### 3.2 Delphi method

The delphi method was a research approach that gathers expert opinions through multiple rounds of feedback, widely used in fields requiring expert knowledge and judgment, particularly for complex issues with high uncertainty [55]. This study is based on a rigorously selected project. The project conducted a rigorous selection process across china, identifying 20 outstanding experts from over 200 candidates. These experts come from various fields such as ceramic craftsmanship, jewelry design, shipbuilding, and sculpture, and possess substantial professional expertise in their respective areas. Additionally, the project focused on offline specialized training in macau’s shipbuilding craftsmanship and portuguese tile painting, and the experts demonstrated a deep understanding of the research topics. In August 2024, the 20 experts were invited to participate in a face-to-face questionnaire survey, with all experts providing verbal informed consent to participate. Experts were required to evaluate 34 items, with each item rated for importance (1–5) and familiarity (0.2–1.0), along with the rationale behind their judgments [56]. The survey sought the experts’ familiarity, judgment basis, and recommendations. Feedback was gradually collected over two rounds to ensure the accuracy of the results. Data analysis was conducted using excel and SPSS software. The authority of the experts was calculated through the arithmetic mean of the judgment basis coefficient (Ca) and the familiarity coefficient (Cs), resulting in an authority coefficient (Cr), with higher Cr values indicating stronger authority [57]. Consistency in expert opinions was measured using the coefficient of variation (CV) and the coordination coefficient (Kendall’s W). A lower CV indicates higher consistency, and a Kendall’s W closer to 1 indicates greater coordination among expert opinions [58].

### 3.3 The DEMATEL-ISM approach

The DEMATEL-ISM method was a comprehensive approach that combines causal analysis and hierarchical modeling to effectively determine causal relationships between factors and identify the most significant key influencing factors [59]. The decision-making trial and evaluation laboratory (DEMATEL) method was a graph theory and matrix-based approach widely used to analyze causal relationships and identify key influencing factors within complex systems [60]. By constructing a causal relationship network among factors, this method reveals the interactions and influence paths between factors, distinguishing core and indirect factors [61]. The interpretive structural modeling (ISM) method, on the other hand, organizes factors into a hierarchical structure based on their relationships, using hierarchical calculations to arrange factors from bottom to top in a logical sequence [62].

In this study, the DEMATEL method primarily identifies and quantifies the causal relationships among key factors. A questionnaire was developed based on factors identified in preliminary research and distributed in November 2024 to experts with over 10 years of teaching experience in traditional craftsmanship. A total of 10 experts agreed to participate. The experts were asked to rate the pairwise influence relationships between indicators on a scale from 0 to 4. The DEMATEL method is used to analyze the logical relationships among the factors in the system, calculating each factor’s degree of influence, degree of being influenced, centrality, and causality. Additionally, the ISM model is employed to identify the intrinsic connections among the influencing factors and establish the hierarchical relationships between them [63]. Through the reachability matrix calculation, factors were arranged hierarchically based on their driving and dependent relationships, thus revealing the hierarchical structure of factors within the system [64].

### 3.4 Research process

**Fig 3:**
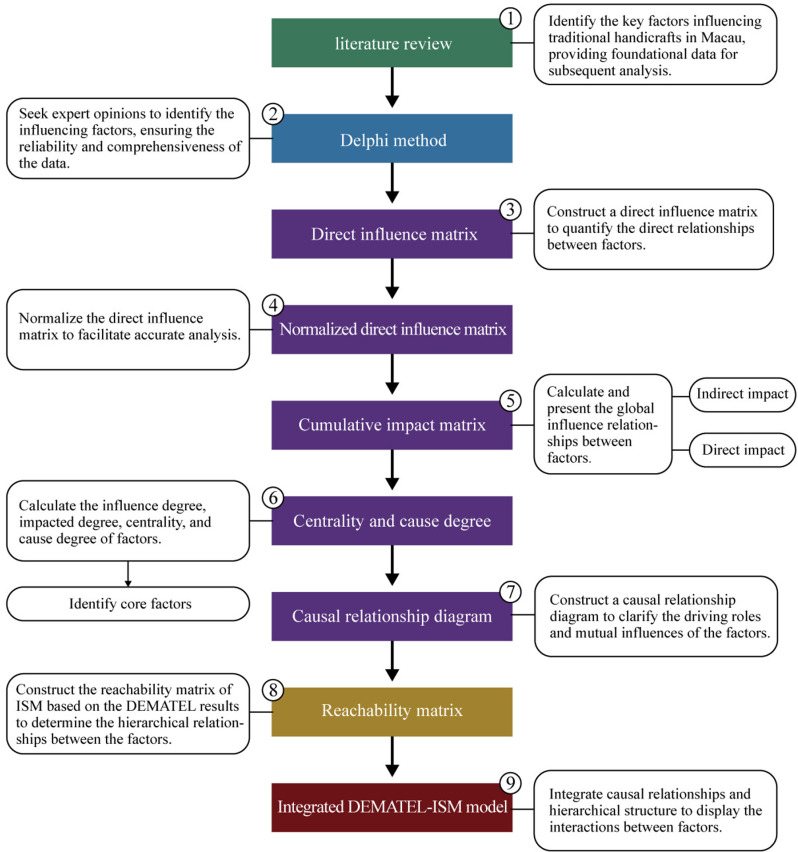
Research flowchart.

This study integrates the delphi method, DEMATEL method, and ISM method. This combination effectively overcomes the limitations of traditional research methods in the analysis of complex systems and provides an in-depth, multidimensional perspective to reveal the multiple factors influencing traditional craftsmanship and their internal relationships. The delphi method plays a foundational role in this research, primarily used for gathering expert opinions, identifying research factors, and constructing causal models [65]. Through two rounds of expert questionnaires, we were able to analyze aspects such as the cultural value and innovative potential of craftsmanship and identify the key elements among the influencing factors. After identifying the key factors through the delphi method, the study further employs the DEMATEL method to analyze the causal relationships among these factors [66]. By constructing a comprehensive influence matrix, it not only reveals the direct and indirect effects between the factors but also quantifies the degree of influence each factor has on others, thereby clearly identifying which factors play a critical role in the innovation and inheritance of traditional craftsmanship in Macau. Building on the results of the DEMATEL method, the study then applies the ISM method. The key function of the ISM method is to transform complex causal relationships into a clear hierarchical structure [67]. By constructing the reachability matrix and conducting hierarchical division, the ISM method helps identify the hierarchical relationships among the factors, demonstrating the levels of influence within the system. This further clarifies the interactions and hierarchical relationships of factors in the innovation and inheritance of traditional craftsmanship in macau. The detailed research process flowchart is shown in Fig 3.

## 4 Research results

### 4.1 Delphi expert questionnaire results

In the first round of the questionnaire, there were 10 experts, with 50% male and 50% female, 60% holding master’s degrees, and 20% and 40% holding associate professor and mid-level titles, respectively. Most had 10–15 years of experience in handicrafts (60%). In the second round, the 10 experts included 40% male and 60% female, with 40% holding doctoral degrees. The distribution of academic ranks was 10% professors, 20% associate professors, and 20% mid-level titles. Experts with 5–9 years of experience in handicrafts accounted for 40%. The basic information of the experts is shown in Table 2. In the first round, the experts’ judgment basis, familiarity, and authority coefficients (Cr) were 0.85, 0.88, and 0.865, respectively. In the second round, these indices improved to 0.91, 0.86, and 0.885, showing an overall improvement in the evaluations. Specific comments on the factor content provided by the experts are shown in Table 3.

**Table 2:** Basic information of experts.

Survey content	Round 1 (n = 10)	Round 2 (n = 10)
Gender		
Male	5	4
Female	5	6
Age		
30-39	9	7
40-49	1	3
50-59		
Education Level		
Bachelor’s	1	1
Master’s	6	5
Doctorate	3	4
Academic Title		
Professor		1
Associate Professor	2	2
Intermediate	4	3
Junior	3	2
Other (e.g., inheritors, artistic directors)	1	2
Years of Involvement in Crafts		
5–9 years	2	4
10–15 years	6	3
16–20 years	2	1
21–25 years		2

**Table 3:** Summary table of importance scores from two rounds of expert consultation questionnaires.

Indicator factors	Indicator title	Round 1				Round 2			
		M	SD	CV	FMF	M	SD	CV	FMF
Historical continuity	Macau shipbuilding crafts/ portuguese tile painting crafts evoke a strong sense of historical culture.	4.40/4.50	0.52/0.71	0.12/0.16	0.27/0.40	3.80/4.20	0.92/0.63	0.24/0.15	0.23/0.23
Local culture	Macau shipbuilding crafts/ portuguese tile painting crafts reflect the uniqueness of macau’s local culture.	4.30/4.60	0.82/0.70	0.19/0.15	0.33/0.47	4.20/4.50	0.79/0.71	0.19/0.16	0.31/0.38
Local craft	Macau shipbuilding crafts/ portuguese tile painting crafts incorporate unique local (macau) techniques.	–	–	–	–	–	–	–	–
Local identity expression	Macau shipbuilding crafts/ portuguese tile painting crafts need to express local cultural identity.	3.60/4.40	0.70/0.70	0.19/0.16	0.07/0.33	3.80/4.80	0.63/0.42	0.17/0.09	0.08/0.62
Cultural pride	Macau shipbuilding crafts/ portuguese tile painting crafts evoke a sense of cultural pride and belonging.	4.30/3.50	0.48/1.08	0.11/0.31	0.20/0.13	–	–	–	–
Storytelling and symbolism	Macau shipbuilding crafts/ portuguese tile painting crafts convey specific cultural stories or symbolic meanings.	3.90/4.40	0.88/0.97	0.22/0.22	0.20/0.47	4.60/4.70	0.70/0.68	0.15/0.14	0.54/0.62
Cultural diversity	Combining diverse cultural elements in Macau shipbuilding crafts/ portuguese tile painting crafts is important	3.40/4.50	0.84/0.53	0.25/0.12	0.07/0.33	3.80/3.80	0.92/0.92	0.24/0.24	0.15/0.23
Integration of tradition and modernity	Integration of traditional and modern design elements in Macau shipbuilding crafts/ portuguese tile painting crafts.	4.10/4.80	0.99/0.42	0.24/0.09	0.33/0.53	4.10/4.40	0.74/0.52	0.18/0.12	0.15/0.31
Creative expression	Macau shipbuilding crafts/ portuguese tile painting crafts need to reflect unique design creativity.	4.10/4.70	0.88/0.48	0.21/0.10	0.27/0.47	4.40/4.50	0.70/0.71	0.16/0.16	0.38/0.46
Craft innovation	Macau shipbuilding crafts/ portuguese tile painting crafts need to showcase innovative capabilities.	–	–	–	–	–	–	–	–
Visual appeal	Macau shipbuilding crafts/ portuguese tile painting crafts have visual appeal.	4.60/4.70	0.52/0.48	0.11/0.10	0.40/0.47	4.10/4.60	0.88/0.70	0.21/0.15	0.31/0.54
Design originality	Macau shipbuilding crafts/ portuguese tile painting crafts’ designs are original.	4.40/4.60	0.84/0.70	0.19/0.15	0.40/0.47	4.10/4.30	0.74/0.68	0.18/0.16	0.23/0.31
Knowledge transmission	Macau shipbuilding crafts/ portuguese tile painting crafts need to effectively convey cultural connotations.	4.40/4.50	0.70/0.53	0.16/0.12	0.33/0.33	3.70/4.20	0.82/0.79	0.22/0.19	0.15/0.31
Craft education	Macau shipbuilding crafts/ portuguese tile painting crafts can inspire interest in learning traditional crafts.	4.50/4.40	0.71/0.84	0.16/0.19	0.40/0.40	3.60/4.00	0.70/0.82	0.19/0.20	0.08/0.23
Emotional communication	Macau shipbuilding crafts/ portuguese tile painting crafts inspire emotional communication and personal experience.	3.80/3.80	0.79/0.63	0.21/0.17	0.13/0.07	4.50/4.50	0.71/0.53	0.16/0.12	0.46/0.38
Emotional experience	Macau shipbuilding crafts/ portuguese tile painting crafts evoke feelings of relaxation and comfort.	3.60/3.70	1.35/0.95	0.38/0.26	0.20/0.13	–	–	–	–
Stimulating reflection	Macau shipbuilding crafts/ portuguese tile painting crafts inspire reflection, deepening the understanding of life or art.	4.10/4.10	0.74/0.57	0.18/0.14	0.20/0.13	3.70/4.20	0.68/0.63	0.18/0.15	0.08/0.23
		M	SD	Threshold		M	SD	Threshold	
Metaphorical approach		4.10/4.35	0.21/0.20	3.89/4.15		4.03/4.36	0.09/0.13	3.94/4.23	
Arithmetic mean		0.20/0.16	0.07/0.06	0.26/0.22		0.19/0.16	0.03/0.04	0.22/0.20	
Coefficient of variation (CV)		0.25/0.34	0.11/0.15	0.14/0.19		0.24/0.37	0.15/0.14	0.09/0.23	

Note: “-” indicates that the item was not covered in that round of the survey.

In the first round, the Kendall’s W was 0.22, χ² = 64.1, p < .001. Experts highly rated both crafts for “historical continuity” and “local culture.” Regarding historical continuity, Macau shipbuilding craftsmanship scored 4.40, and portuguese tile painting scored 4.50. In the local culture dimension, the scores were 4.30 and 4.60, respectively, showing the experts’ strong recognition of their historical and cultural values. Some experts argued that macau’s shipbuilding craftsmanship is more of a symbol of fishing history. While it has gradually faded from functional use, it still holds historical value. Portuguese tile painting, although considered an external cultural influence, has become part of macau’s visual culture, particularly in religious art and public spaces, symbolizing portuguese colonial culture. Some experts pointed out that the cultural identity of macau’s shipbuilding craftsmanship is weaker, mainly identified by fishermen or artisan communities, rather than the broader public. Portuguese tile painting bears clear Portuguese cultural symbols but has not fully integrated Macau’s local cultural elements, failing to present macau’s unique cultural identity entirely. Most experts agreed that both crafts performed well in terms of local identity expression, storytelling and symbolism, and creative expression. Overall, experts gave positive evaluations for their visual appeal, design originality, and knowledge transmission.

However, some indicators did not gain unanimous recognition and were deleted. For example, “local craft” faced objections from four experts, who argued that it was too broad or did not align with the current connotations of handicrafts, leading to its removal. “cultural pride” and “emotional experience” had CVs of 0.31/0.26, exceeding the preset threshold, showing significant disagreement, and were also deleted. Two experts raised concerns about the lack of clarity regarding the direction of innovation in craftsmanship, failing to distinguish between innovation in outcomes and processes. Due to the limitations of traditional techniques, Macau’s shipbuilding craftsmanship struggles to integrate multiple cultural elements, and experts generally felt that it should maintain its traditional character.

Experts unanimously agreed that there is limited space for innovation in traditional handicrafts and suggested a greater focus on the practicality of the craft. Therefore, these elements were also deleted. The first-round results showed that experts strongly recognized the cultural heritage and historical value of these crafts, but there was considerable divergence in their evaluations regarding innovation and emotional experience. Experts generally believed that creative expression in macau’s shipbuilding craftsmanship is limited, mainly focused on technical details rather than major structural or functional changes. Excessive incorporation of modern design elements could dilute its historical and cultural significance. On the other hand, portuguese tile painting demonstrated greater potential for modernization, capable of incorporating modern elements in patterns, materials, and application scenarios, attracting younger audiences and achieving both cultural communication and commercialization goals.

In the second round, the Kendall’s W was 0.21, χ² = 59.6, p < .001. In terms of cultural and historical value, experts still gave high scores for “historical continuity” and “local culture,” with scores of 3.80/4.20 and 4.20/4.50, indicating the importance of these dimensions. Experts’ evaluations showed minimal variation, with CVs all below 0.25, suggesting a high level of agreement. Portuguese tile painting received high ratings for local identity expression, storytelling and symbolism, and creative expression. Macau shipbuilding craftsmanship scored lower in cultural diversity and craft education. Due to its complexity and high technical barriers, macau’s shipbuilding craftsmanship faces certain limitations in popularization and educational promotion. Experts suggested that exhibitions and courses could help the public better understand its cultural connotations and technical essence. In contrast, Portuguese tile painting, with its relatively simple learning process, is particularly suitable for school education, stimulating students’ artistic interest and cultural identity. Many experts noted that learning this craft is not only physical labor but also a spiritual enjoyment, offering deep emotional experiences.

Experts unanimously recognized both crafts for their emotional communication and the blending of tradition and modernity. They also gave positive evaluations for cultural content transmission and creative expression. Finally, after two rounds of expert questionnaires, the following modifications were made: indicators with significant disagreement, such as “local craft,” “cultural pride,” “emotional experience,” and “craft innovation,” were deleted. The newly added “practicality of the craft” received greater attention, and 14 indicators were retained (see Table 4).

**Table 4:** Example of expert interview opinions.

Element	Expert opinion (macau shipbuilding crafts)	Expert opinion (portuguese tile painting crafts)
Historical continuity	B9 believes it is like other coastal regions, A10 believes functionality is gradually being phased out, A5 believes the technique continues the fishing history.	B9 believes there are few elements reflecting Macau’s culture, mostly reflecting Portuguese culture.
Local culture	A10 believes there is no obvious difference from the Pearl River Delta, B9 emphasizes local craftsmanship, but relies on the craftsman’s intuition.	A8 believes it shows a strong sense of historical culture and religious art.
Local identity expression	B5 believes the symbolic nature is low, A2 believes there is a lack of official support.	A8 believes it is a symbol of foreign culture, B5 believes it is closely tied to Portuguese culture.
Storytelling and symbolism	A4, A10 believe the cultural story mainly relies on oral tradition, with limited dissemination effect.	A8 believes it has symbolic religious stories and cultural exchange.
Cultural diversity	A2 believes traditional crafts limit cultural integration, B7 believes the core value lies in maintaining tradition.	Most experts generally believe there is great potential for cultural integration, A4 also believes it is suitable for multicultural innovation.
Integration of tradition and modernity	A1, A3 believe tradition should be preserved, A10 believes modern design focuses on functionality.	A8, B6 believe integrating modern design can attract young audiences, A4 believes it can promote cultural dissemination.
Creative expression	B8 believes the core value lies in technique, A6 believes creativity should be reflected in communication rather than changes in craftsmanship.	A4, A7 believe it should embody design creativity, A3 believes it can integrate Macau’s cultural characteristics.
Craft practicality	B7, A9 believe craftsmanship is reflected in technical details, A1 believes the practicality of the craft should be emphasized.	A5, A8 believe there is considerable potential for innovation, A3 believes modern technology should be integrated to enhance performance and practicality.
Visual appeal	A3 believes aesthetic value lies in structural beauty, B6 believes the appeal is weak.	A7, A10 believe it has high visual appeal, B5 believes its decorative and artistic value is important.
Design originality	B9, B7 believe originality is not important, A6 believes tradition is already fixed.	B8 believes originality is limited, B7 believes originality is needed to attract attention, B4 believes originality is not important.
Knowledge transmission	A8 believes it has cultural educational value, but the dissemination effect is weak, A3 believes it should be spread through courses or exhibitions.	A9, B6 believe it has strong cultural dissemination potential, A4 believes it is suitable for school education.
Craft education	A7 believes complexity limits educational promotion, B9 believes it can spark students’ interest.	A6 believes it is suitable for educational promotion, A5 believes it can cultivate cultural identity.
Emotional communication	A3 believes emotional communication is established through interaction and collaboration with craftsmen, B8 believes it is established through labor experiences.	A4 believes emotional communication is reflected in the creator’s artistic expression, B7 believes it is limited.
Stimulating reflection	A3 believes it can inspire reflection on life and art, A1 believes it inspires reflection on the value of craftsmanship.	A9 believes it can inspire artistic creation, B6 believes it has limited reflection on cultural symbolism.

### 4.2 DEMATEL decision analysis results

**Step 1: Direct influence matrix**

Based on the factors influencing traditional handicrafts identified in the previous research, a questionnaire survey was conducted with 10 traditional handicraft experts (50% male, 50% female, including university professors and artisans). Each expert rated the relationships between the influence factors on a scale of 0, 1, 2, 3, and 4. After averaging the experts’ scores, the direct influence matrix A was obtained, as shown in Table 5. By comparing the magnitude of influence, it was found that factors do not influence themselves; therefore, the diagonal elements of the matrix were assigned a value of 0. The direct influence matrix A was constructed using the formula (1) [68].

**Table 5:** Direct influence matrix A.

Factor	A1	A2	B1	B2	B3	C1	C2	C3	D1	D2	E1	E2	F1	F2
A1	0.00	3.00	3.00	3.40	4.00	2.60	2.70	2.50	3.00	2.40	4.00	1.00	3.00	4.00
A2	3.00	0.00	3.10	1.00	3.00	2.60	2.90	1.00	2.60	4.00	2.60	2.50	3.60	3.20
B1	2.90	2.80	0.00	3.40	1.00	2.60	2.60	2.20	2.10	2.60	2.70	2.40	3.50	3.20
B2	2.60	3.00	2.80	0.00	3.00	2.70	3.10	1.80	2.70	2.80	1.00	2.20	2.90	2.90
B3	2.50	2.40	0.00	2.80	0.00	2.90	3.10	2.70	3.00	0.00	3.00	2.90	3.20	1.00
C1	2.80	2.90	3.00	3.30	1.00	0.00	4.00	2.90	3.20	3.30	4.00	2.90	2.70	3.20
C2	1.80	2.00	2.60	3.00	3.20	3.50	0.00	3.30	3.60	3.60	2.60	2.50	2.80	3.20
C3	2.20	4.00	2.00	4.00	2.40	2.70	2.90	0.00	2.20	3.00	1.00	2.80	2.30	1.00
D1	2.10	2.10	2.20	2.80	2.90	2.80	3.30	2.60	0.00	3.50	2.40	2.40	3.20	3.10
D2	1.90	2.50	2.40	3.00	2.90	4.00	3.30	2.60	3.00	0.00	2.70	0.00	2.90	3.50
E1	3.30	3.30	3.20	1.00	3.30	3.10	3.20	2.90	2.70	2.60	0.00	3.40	3.20	3.20
E2	1.00	4.00	1.00	2.90	2.00	2.80	2.60	0.00	2.00	2.60	2.90	0.00	2.80	2.80
F1	3.40	3.20	3.60	3.40	3.10	2.60	2.70	1.80	2.80	2.60	2.80	2.60	0.00	2.80
F2	2.00	2.30	2.10	2.60	4.00	2.50	2.90	2.30	4.00	3.10	2.60	1.00	2.30	0.00

Note: Historical continuity = A1, Local culture = A2, Local identity expression = B1, Storytelling and symbolism = B2, Cultural diversity = B3, Integration of tradition and modernity = C1, Creative expression = C2, Craft practicality = C3, Visual appeal = D1, Design originality = D2, Knowledge transmission = E1, Craft education = E2, Emotional communication = F1, Stimulating reflection = F2.


$$A=[\begin{array}{ccc} 0 & \cdots & x_{1n}\\ \vdots & \ddots & \vdots \\ x_{n1} & \cdots & 0 \end{array}]$$ (1)

**Step 2: Normalized influence matrix**

By calculating the sum of the elements in each row of matrix A, the maximum row sum was selected as the baseline value. The elements of matrix Awere then normalized using this baseline value [68], resulting in the normalized influence matrix B, as shown in Table 6.

**Table 6:** Normalized influence matrix B.

Factor	A1	A2	B1	B2	B3	C1	C2	C3	D1	D2	E1	E2	F1	F2
A1	0.00	0.08	0.08	0.09	0.10	0.07	0.07	0.06	0.08	0.06	0.10	0.03	0.08	0.10
A2	0.08	0.00	0.08	0.03	0.08	0.07	0.07	0.03	0.07	0.10	0.07	0.06	0.09	0.08
B1	0.07	0.07	0.00	0.09	0.03	0.07	0.07	0.06	0.05	0.07	0.07	0.06	0.09	0.08
B2	0.07	0.08	0.07	0.00	0.08	0.07	0.08	0.05	0.07	0.07	0.03	0.06	0.07	0.07
B3	0.06	0.06	0.00	0.07	0.00	0.07	0.08	0.07	0.08	0.00	0.08	0.07	0.08	0.03
C1	0.07	0.07	0.08	0.08	0.03	0.00	0.10	0.07	0.08	0.08	0.10	0.07	0.07	0.08
C2	0.05	0.05	0.07	0.08	0.08	0.09	0.00	0.08	0.09	0.09	0.07	0.06	0.07	0.08
C3	0.06	0.10	0.05	0.10	0.06	0.07	0.07	0.00	0.06	0.08	0.03	0.07	0.06	0.03
D1	0.05	0.05	0.06	0.07	0.07	0.07	0.08	0.07	0.00	0.09	0.06	0.06	0.08	0.08
D2	0.05	0.06	0.06	0.08	0.07	0.10	0.08	0.07	0.08	0.00	0.07	0.00	0.07	0.09
E1	0.08	0.08	0.08	0.03	0.08	0.08	0.08	0.07	0.07	0.07	0.00	0.09	0.08	0.08
E2	0.03	0.10	0.03	0.07	0.05	0.07	0.07	0.00	0.05	0.07	0.07	0.00	0.07	0.07
F1	0.09	0.08	0.09	0.09	0.08	0.07	0.07	0.05	0.07	0.07	0.07	0.07	0.00	0.07
F2	0.05	0.06	0.05	0.07	0.10	0.06	0.07	0.06	0.10	0.08	0.07	0.03	0.06	0.00

**Step 3: Comprehensive influence matrix**

The comprehensive influence matrix reflects the overall impact of interactions among the elements within the system (see Table 7). The calculation method is as shown in formula (2) [69].

**Table 7:** Comprehensive influence matrix T.

Factor	A1	A2	B1	B2	B3	C1	C2	C3	D1	D2	E1	E2	F1	F2
A1	0.54	0.70	0.61	0.69	0.71	0.70	0.73	0.56	0.70	0.67	0.68	0.52	0.72	0.72
A2	0.57	0.57	0.57	0.59	0.63	0.64	0.68	0.48	0.64	0.66	0.60	0.51	0.68	0.66
B1	0.55	0.63	0.48	0.63	0.57	0.63	0.66	0.50	0.62	0.61	0.59	0.50	0.66	0.64
B2	0.53	0.62	0.53	0.54	0.61	0.62	0.65	0.48	0.62	0.60	0.54	0.48	0.64	0.62
B3	0.48	0.55	0.42	0.55	0.48	0.56	0.59	0.45	0.56	0.48	0.53	0.46	0.58	0.52
C1	0.62	0.71	0.62	0.71	0.65	0.65	0.77	0.58	0.72	0.71	0.69	0.57	0.73	0.72
C2	0.57	0.66	0.58	0.67	0.67	0.70	0.65	0.56	0.70	0.68	0.63	0.54	0.70	0.69
C3	0.51	0.62	0.50	0.61	0.57	0.60	0.63	0.42	0.59	0.59	0.52	0.48	0.60	0.56
D1	0.55	0.63	0.55	0.63	0.63	0.65	0.69	0.52	0.58	0.65	0.60	0.51	0.67	0.65
D2	0.54	0.63	0.55	0.63	0.63	0.67	0.69	0.52	0.65	0.56	0.60	0.45	0.66	0.66
E1	0.62	0.70	0.61	0.64	0.68	0.70	0.74	0.56	0.69	0.67	0.58	0.57	0.72	0.70
E2	0.45	0.58	0.44	0.54	0.53	0.56	0.58	0.39	0.54	0.54	0.53	0.38	0.57	0.56
F1	0.61	0.68	0.60	0.68	0.67	0.68	0.71	0.53	0.68	0.66	0.63	0.54	0.63	0.68
F2	0.53	0.60	0.52	0.60	0.63	0.62	0.65	0.50	0.65	0.61	0.58	0.46	0.63	0.55


$$T=\left(B+B^{2}+\cdots +B^{k}\right)=B(I-B{)}^{-1}$$ (2)

**Step 4: Calculation of centrality and causality**

**Fig 4:**
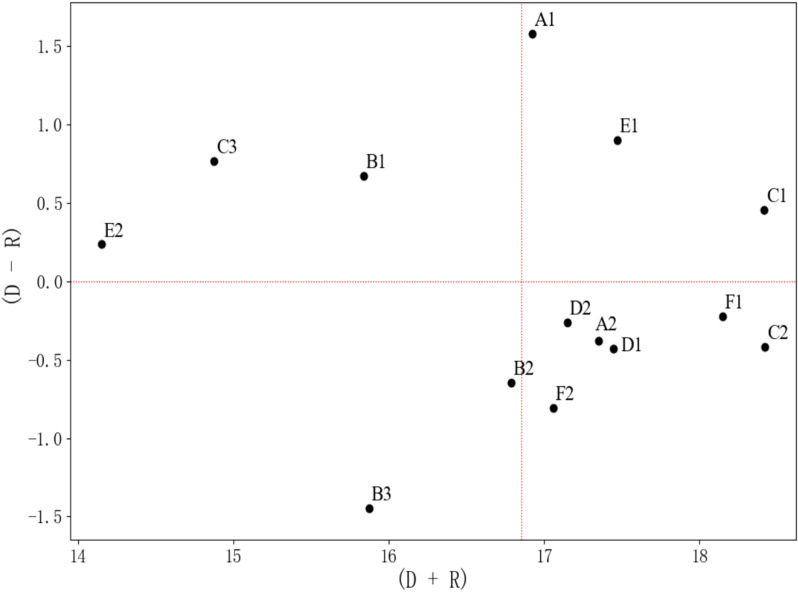
Relationship between centrality (D + C) and causality (D-C).


Using the comprehensive influence matrix, the influence degree, being influenced degree, centrality, and causality of each factor in the system were determined, as shown in Table 8. The calculation formulas are as follows [68]. The relationship between centrality (D + C) and causality (D - C) is illustrated in Fig 4.

**Table 8:** DEMATEL analysis results of cultural identity’s influence on traditional craft.

Factor	Factor name	(D)	(C)	(D + C)	(D-C)	Weight	Centrality ranking	Factor attribute
A1	Historical continuity	9.25	7.67	16.92	1.58	0.07	9	Cause factor
A2	Local culture	8.49	8.86	17.35	-0.38	0.07	6	Result factor
B1	Local identity expression	8.26	7.58	15.84	0.67	0.07	12	Cause factor
B2	Storytelling and symbolism	8.07	8.72	16.79	-0.65	0.07	10	Result factor
B3	Cultural diversity	7.21	8.66	15.87	-1.45	0.07	11	Result factor
C1	Integration of tradition and modernity	9.44	8.98	18.42	0.46	0.08	2	Cause factor
C2	Creative expression	9.00	9.42	18.42	-0.42	0.08	1	Result factor
C3	Craft practicality	7.82	7.05	14.88	0.77	0.06	13	Cause factor
D1	Visual appeal	8.51	8.94	17.45	-0.43	0.07	5	Result factor
D2	Design originality	8.44	8.71	17.15	-0.26	0.07	7	Result factor
E1	Knowledge transmission	9.18	8.28	17.47	0.90	0.07	4	Cause factor
E2	Craft education	7.19	6.96	14.15	0.24	0.06	14	Cause factor
F1	Emotional communication	8.96	9.19	18.15	-0.22	0.08	3	Result factor
F2	Stimulating reflection	8.12	8.93	17.06	-0.81	0.07	8	Result factor

$$D_{i}=\sum_{j=1}^{n}x_{ij},(i=1,2,\ldots ,n)$$ (3)

$$C_{i}=\sum_{j=1}^{n}x_{ji},(i=1,2,\ldots ,n)$$ (4)

$$M_{i}=D_{i}+C_{i}$$ (5)

$$R_{i}=D_{i}-C_{i}$$ (6)

In Table 8, the DEMATEL analysis results of the influencing factors of cultural identity on traditional handicrafts are presented, including key indicators such as influence degree, being influenced degree, centrality, causality, weight, centrality ranking, and factor attributes [70].

The influence degree reflects the overall impact of a factor on other factors. Factors C1 and E1 exhibit a strong driving force and significantly impact other factors in the system. On the other hand, the being influenced degree reveals the extent to which a factor is affected by other factors. F1 and C2 have the highest being influenced degree, indicating that they are more susceptible to the influence of other factors in the system.

Centrality is the sum of influence degree and being influenced degree, representing the overall importance of a factor in the system. Both C1 and C2 have a centrality of 18.42, ranking at the top, which indicates their central role in the system. In contrast, C3 and E2 have the lowest centrality, suggesting that their importance in the system is relatively weak.

From the causality analysis, factors with positive values, such as A1 and E1, are key driving factors in the system, exerting strong influence. Conversely, factors with negative values, such as B3 and F2, are outcome factors and indirect factors, being more influenced by other factors. This clear distinction between direct and indirect factors effectively reflects the intrinsic mechanisms within the system.

In the factor attribute ranking, C1 and C2 rank 2nd and 1st, respectively, due to their central roles, while E2 and C3 are ranked lower, indicating their relatively limited contribution to the system.

### 4.3 ISM model results evaluation

**Fig 5:**
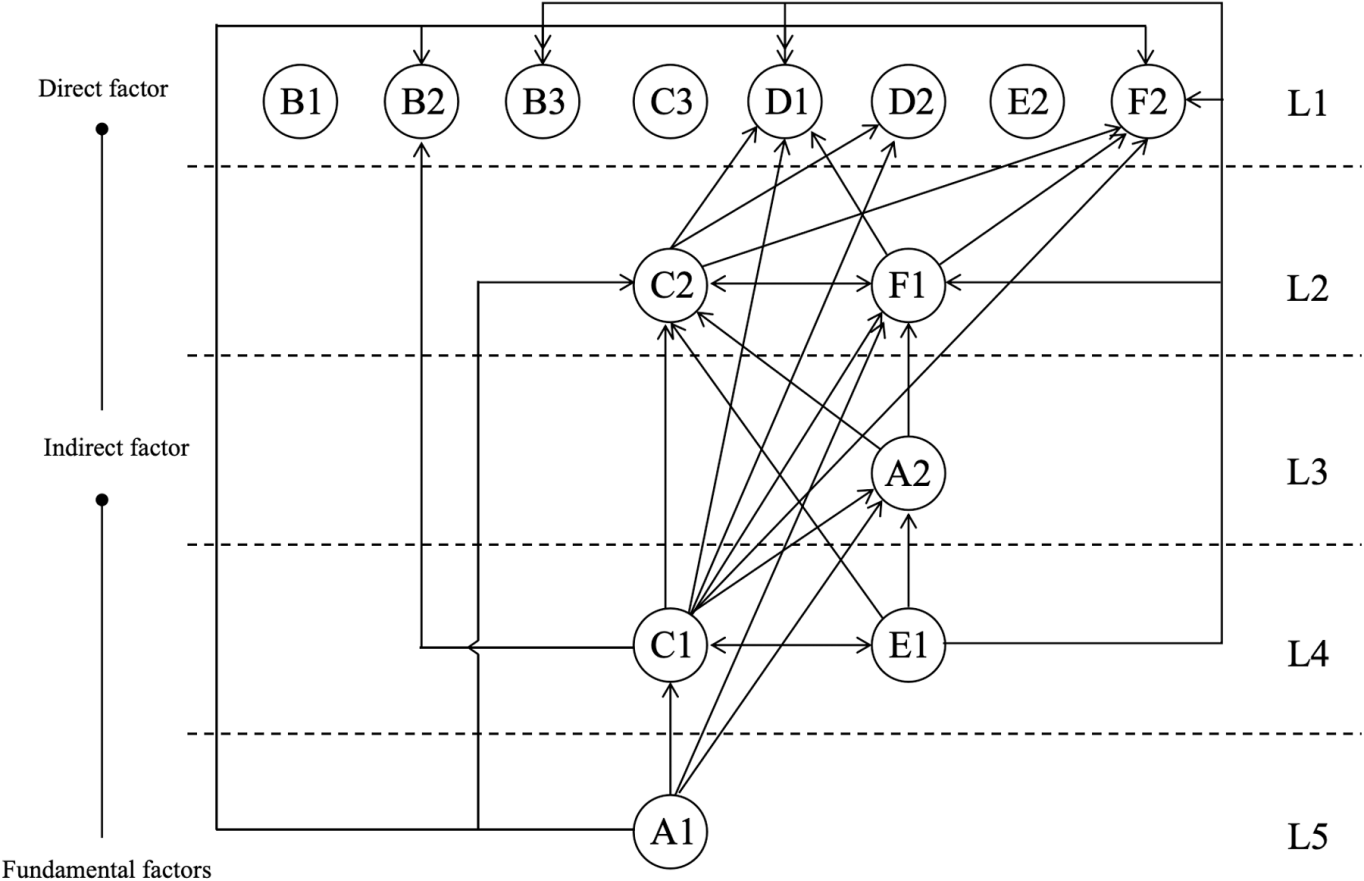
Hierarchical factor classification model.

Based on the DEMATEL analysis, the ISM method is further employed to analyze the influencing factors and reveal the hierarchical structure of the system. The construction of the reachability matrix is an important step in analyzing the system’s hierarchical structure [71]. Table 9 shows the reachability matrix calculated based on the comprehensive influence matrix, with the threshold λ selected as the sum of the mean and standard deviation of the comprehensive influence matrix [72]. Based on this, the positions of the different influencing factors are ranked, and a hierarchical model (Fig 5) is established to clarify the direct and indirect pathways of influence in the system.

**Table 9:** Reachability matrix.

Factor	A1	A2	B1	B2	B3	C1	C2	C3	D1	D2	E1	E2	F1	F2
A1	1	1	0	1	1	1	1	0	1	0	0	0	1	1
A2	0	1	0	0	0	0	1	0	0	0	0	0	1	0
B1	0	0	1	0	0	0	0	0	0	0	0	0	0	0
B2	0	0	0	1	0	0	0	0	0	0	0	0	0	0
B3	0	0	0	0	1	0	0	0	0	0	0	0	0	0
C1	0	1	0	1	0	1	1	0	1	1	1	0	1	1
C2	0	0	0	0	0	1	1	0	1	1	0	0	1	1
C3	0	0	0	0	0	0	0	1	0	0	0	0	0	0
D1	0	0	0	0	0	0	1	0	1	0	0	0	0	0
D2	0	0	0	0	0	0	1	0	0	1	0	0	0	0
E1	0	1	0	0	1	1	1	0	1	0	1	0	1	1
E2	0	0	0	0	0	0	0	0	0	0	0	1	0	0
F1	0	1	0	0	0	0	1	0	1	0	0	0	1	1
F2	0	0	0	0	0	0	0	0	0	0	0	0	0	1

In the hierarchical factor classification model, five levels (L1 to L5) clearly reflect the hierarchical relationships and influence pathways of the factors in the system.

- Level L5 (fundamental layer) contains A1, the core factor that serves as the driving force of the system. A1 influences all other factors in the upper layers, either directly or indirectly, and is the foundational power source for the entire system.
- Level L4 contains C1 and E1, which act as key bridging factors that directly transmit A1’s driving force to the intermediate and surface layers, playing a crucial role in linking the upper and lower levels of the system.
- Level L3 consists of A2, which serves as a supporting factor in the system, linking the higher layers C1 and E1 with the intermediate layer, while providing support to the factors in Level L2.
- Level L2 is composed of C2 and F1, which serve as intermediary transitional factors that are both driven by the upper layers and exert significant influence on the surface layer (L1). They function as bridges between the middle layers and the surface output.
- Level L1 (surface layer) includes B1, B2, B3, C3, D1, D2, E2, and F2, which are indirect factors representing the final manifestations of the system, mainly influenced by the combined effects of the upper layers.

From the perspective of the influence pathways, A1 directly influences C1 and indirectly drives the entire system’s dynamic changes through these key direct factors. Both C1 and E1 are not only core mediating factors driven by A1, but they also significantly affect the intermediate layer (A2) and the surface layer (L1), making them essential transmission nodes within the system. C2 and F1, as intermediate transitional layers, transmit the effects of the upper layers to the surface and drive the changes in indirect factors such as D1, D2, and F2.

Through the analysis of the reachability matrix, the antecedent set, reachable set, intersection set, and hierarchical classification of each factor are generated. Table 10 clearly reveals the hierarchical structure and influence pathways of the factors in the system. The analysis of the reachability set, antecedent set, and intersection set shows the interrelationships between the factors and the dynamic connections between the levels, providing clear theoretical and practical references for system optimization and intervention strategies.

**Table 10:** Antecedent and reachable sets.

Factor	Reachability set	Antecedent set	Intersection set	Level
A1	A1, A2, B2, B3, C1, C2, D1, F1, F2	A1	A1	L5
A2	A2, C2, F1	A1, A2, C1, E1, F1	A2, F1	L3
B1	B1	B1	B1	L1
B2	B2	A1, B2, C1	B2	L1
B3	B3	A1, B3, E1	B3	L1
C1	A2, B2, C1, C2, D1, D2, E1, F1, F2	A1, C1, C2, E1	C1, C7, E1	L4
C2	C1, C2, D1, F1, F2	A1, A2, C1, C2, D1, D2, E1, F1	C1, C2, D1, D2, F1	L2
C3	C3	C3	C3	L1
D1	C2, D1	A1, C1, C2, D1, E1, F1	C2, D1	L1
D2	C2, D2	C1, C2, D2	C2, D2	L1
E1	A2, B3, C1, C2, D1, E1, F1, F2	C1, E1	C1, E1	L4
E2	E2	E2	E2	L1
F1	A2, C2, D1, F1, F2	A1, A2, C1, C2, E1, F1	A2, C2, F1	L2
F2	F2	A1, C1, C2, E1, F1, F2	F2	L1

The highest-level core factors play a decisive role in the system’s operation, while the middle and lower-level factors reflect the complex internal interactions and the final outputs of the system. For example, the reachability set of A1 includes A1, A2, B2, B3, C1, C2, D1, F1, and F2, indicating that A1 has a wide range of influence, capable of directly or indirectly affecting multiple factors. The antecedent set represents all the factors that directly or indirectly influence a particular factor. For instance, the antecedent set of C2 includes A1, A2, C1, D1, D2, E1, and F1, showing that these factors significantly influence C2. The intersection set is the overlap between the reachability set and the antecedent set, representing factors that both influence and are influenced by a particular factor. For example, the intersection set of C2 includes C1, C2, D1, D2, and F1, indicating bidirectional influence among these factors.

Based on the analysis of the intersection set, the hierarchy is further refined. The highest level (L5) consists of A1, which is the core factor in the system, exerting a significant influence on other factors in the system. The second-highest level (L4) includes C1 and E1, which play a bridging role between the upper and middle levels. The intermediate level (L3) consists of A2 and F1, which are driven by the higher layers and exert influence on lower-level factors. The lower level (L2) consists of C2, which plays a bridging role in the system. The lowest level (L1) includes B1, B2, B3, C3, D1, D2, E2, and F2, which mainly serve as indirect factors, reflecting the result of the combined effects of factors from other levels.

## 5. Discussions

**Fig 6:**
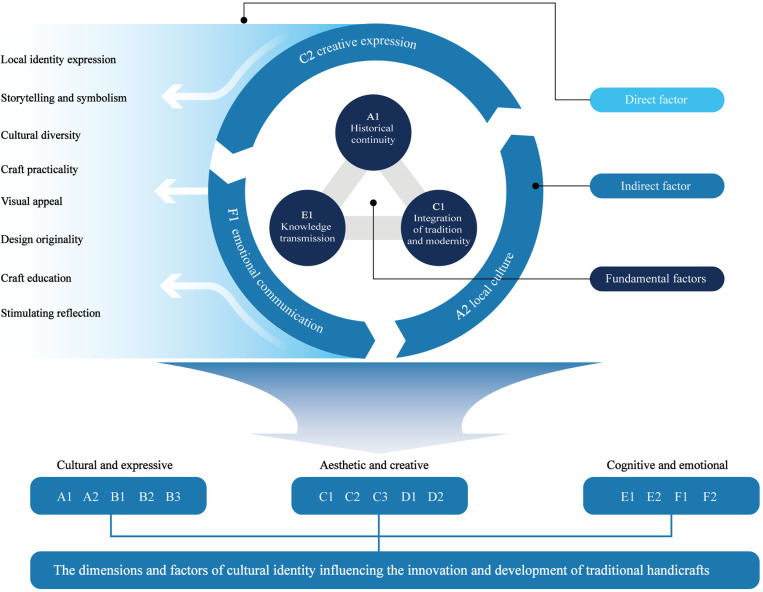
Structural diagram of cultural identity’s impact on traditional craft innovation and evaluation system.

This study, through the hierarchical framework model, systematically reveals the core driving role of cultural identity in the innovation of traditional crafts and its hierarchical influence pathways (results shown in Fig 6). The findings indicate that historical continuity (A1), knowledge transmission (E1), and the integration of tradition and modernity (C1) constitute the core factors, laying the cultural foundation for the innovation of traditional crafts. These factors directly reflect the profound connotations of cultural identity. Among them, historical continuity ensures the continuity of traditional craftsmanship within its historical context [73]. This continuity not only reflects the deep value of cultural heritage but also highlights cultural identity as a key support for traditional crafts; knowledge transmission, through education, dissemination, and practical pathways [74], deepens the public’s understanding and recognition of traditional craftsmanship, providing a continuous driving force for craft innovation; the integration of tradition and modernity, by incorporating modern design language while preserving the core of traditional culture, opens up new spaces for traditional crafts in modern consumer contexts [75]. This core layer not only ensures a deep connection between crafts and culture but also drives innovation in traditional crafts within the contemporary context through a strong sense of cultural identity [76]. To further strengthen the foundational role of cultural identity, efforts should focus on recording the historical context and representative techniques of traditional crafts, establishing digital archives for crafts [77], and showcasing their cultural value to the public through documentaries, exhibitions, performances, and other forms [78]. Additionally, crafting knowledge should be formalized and standardized to ensure systematic and sustainable knowledge transmission [45]. Moreover, supporting design innovation is crucial, encouraging designers to reinterpret traditional crafts using modern languages [12]. For example, integrating modern technologies (such as 3D printing, sustainable materials) and contemporary design aesthetics can make traditional crafts more relevant and competitive in the market [79].

In the framework, direct factors (such as creative expression (C2), local culture (A2), and emotional communication (F1)) inherit the driving force from the core factors and further influence the value manifestation in the indirect layer. These direct factors play a key role in bridging the multidimensional needs of traditional crafts, achieving a balance between cultural heritage and commercial practice. For instance, creative expression, as a direct factor, reflects how designers redefine traditional crafts through innovative forms and modern languages [80]; emotional communication highlights how craft products evoke emotional resonance in consumers through aesthetics, storytelling, or interactivity, directly enhancing their social and market value [81]; local culture emphasizes the unique identity of traditional crafts within regional cultural contexts [15]. These factors are crucial for transforming the cultural value of traditional crafts into social and market value. It is essential to support diverse attempts at creative expression, encouraging collaboration between designers, artists, and artisans, and integrating crafts into modern art installations, fashion design, and digital media [82]. In terms of emotional communication, enhancing interactive experiences (such as open workshops and hands-on craftsmanship experiences) can strengthen emotional resonance [83]. Additionally, incorporating storytelling can give products deeper cultural meanings [84].

The indirect factors further reflect the multidimensional value of traditional crafts in modern society. Factors such as local identity expression (B1), storytelling and symbolism (B2), and cultural diversity (B3) indirectly enhance the cultural identity of traditional crafts by enriching cultural expressions. Craft practicality (C3), visual appeal (D1), and design originality (D2) showcase the dual advantages of traditional crafts in both artistic and market competitiveness. Stimulating reflection (F2) and craft education (E2) reflect the important role of crafts in human emotions and social interactions. For example, Macau’s boat-building technique can enhance emotional connections between consumers and cultural heritage through skill demonstrations and narrative methods. The Portuguese Azulejo tiles, with their historical narrative design, not only satisfy decorative needs but also provoke public reflection on their cultural background, highlighting the dual value of crafts in both art and education.

From an overall structural perspective, the model starts with the cultural foundation and, through core, direct, and indirect factors, builds a complete pathway from cultural identity to innovative practice in traditional crafts. This model illustrates the collaborative role of multidimensional factors in traditional craft innovation. The relationships between different layers are both independent and interwoven, forming a system framework characterized by internal and external interaction and dynamic feedback.

## 6. Theoretical and practical significance

This study combines the DEMATEL-ISM method to provide a new perspective for factor analysis and causal relationship modeling in complex systems. This approach not only reveals the systematic path of traditional craftsmanship innovation and cultural identity but also offers practical strategies for the protection of craftsmanship. Theoretically, the study identifies historical continuity, knowledge transmission, and the integration of tradition and modernity as the core factors driving craftsmanship innovation. This finding underscores the fundamental role of cultural identity in the innovation of craftsmanship, especially in the continuation of traditional skills. Historical continuity ensures the inheritance of traditional crafts within their historical context, preserving their cultural value and strengthening societal recognition of craftsmanship [46,85]. Knowledge transmission, through education, dissemination, and practice, continuously promotes the innovation and development of traditional craftsmanship [86]. This provides direction for future research on the innovation of traditional craftsmanship and further reveals the critical role of cultural identity in this process. Practically, preserving and showcasing the historical context and representative skills of traditional crafts is crucial for enhancing public cultural identity and understanding. Specifically, the establishment of digital archives for craftsmanship and the use of exhibitions, documentaries, and other formats to present the cultural value of traditional crafts will help enhance societal recognition [79,87,88]. Furthermore, integrating modern design language with the core elements of traditional culture can open new avenues for the application of traditional crafts in the contemporary consumer market [89,90]. These practical measures contribute to the transmission and innovation of traditional craftsmanship in modern society.

## 7. Conclusion and limitation

This study, based on the DEMATEL-ISM methodology, constructs a hierarchical framework to systematically analyze the relationship between traditional craft innovation and cultural identity. The research shows that historical continuity, knowledge transmission, and the integration of tradition and modernity are the core factors that lay the cultural foundation for innovation. Direct factors, such as creative expression, local culture, and emotional communication, play a key role in bridging the core and indirect layers, highlighting their pivotal position in innovation practice. Indirect factors, such as visual appeal, design originality, and craft education, further demonstrate the multidimensional effectiveness of traditional crafts in artistic value, cultural dissemination, and social interaction. Cultural identity is not only an important driving force for traditional craft innovation but also the key force in realizing its multidimensional value. These findings provide valuable pathways and strategies for the protection and innovation of traditional crafts.

However, this study has some limitations. The development of traditional crafts is influenced by various factors. Besides cultural identity, factors such as economics, technology, and social changes also play important roles. Future research could further explore how these different factors contribute to craft innovation. Additionally, this study uses the traditional crafts of macau as a case, which, while representative, may require further validation in different regions or cultural contexts. This study provides rich content and in-depth analysis, deepening the understanding of the relationship between traditional craft innovation and cultural identity. It also offers valuable insights and references for future research and practice in craft innovation.

## Supporting information

S1 Appendix Dataset. (XLSX)
